# Syndromic and Non-Syndromic Primary Failure of Tooth Eruption: A Genetic Overview

**DOI:** 10.3390/genes16020147

**Published:** 2025-01-24

**Authors:** Clarissa Modafferi, Elisabetta Tabolacci, Cristina Grippaudo, Pietro Chiurazzi

**Affiliations:** 1 UOC Genetica Medica, Fondazione Policlinico Universitario “A. Gemelli” IRCCS, 00168 Rome, Italy; clarissamodafferi@libero.it (C.M.); pietro.chiurazzi@unicatt.it (P.C.); 2 Dipartimento di Scienze della Vita e Sanità Pubblica, Sezione di Medicina Genomica, Università Cattolica del Sacro Cuore, 00168 Rome, Italy; elisabetta.tabolacci@unicatt.it; 3 Fondazione Policlinico Universitario “A. Gemelli” IRCCS, 00168 Rome, Italy; 4 UOC Clininica Odontoiatrica, Dipartimento di Neuroscienze, Organi di Senso e Torace, Fondazione Policlinico Universitario “A. Gemelli” IRCCS, 00168 Rome, Italy; 5 Odontoiatria e Protesi Dentaria, Dipartimento Testa Collo ed Organi di Senso, Università Cattolica del Sacro Cuore, 00168 Rome, Italy

**Keywords:** PFE, *PTH1R* gene, *TMEM119* gene, isolated PFE, syndromic PFE

## Abstract

Primary failure of tooth eruption (PFE) is a rare genetic disorder characterized by the failure of teeth to erupt in the absence of obvious physical obstructions, often resulting in a progressive open bite that is resistant to orthodontic treatment. While PFE can be caused by genetic or systemic factors (such as cysts, tumors, and endocrine imbalances), the non-syndromic causes are primarily genetic, with an autosomal dominant inheritance pattern with variable expressivity. Several genes have been closely associated with the non-syndromic PFE form. The *PTH1R* (parathyroid hormone 1 receptor) is the most commonly PFE-associated gene. Additional genes associated with minor frequency are Transmembrane protein 119 (*TMEM119*), which reduces the glycolytic efficiency of bone cells, limiting their mineralization capacity and causing bone fragility; Periostin (*POSTN*), which regulates the extracellular matrix and the bone’s response to mechanical stress; and Lysine (K)-specific methyltransferase 2C (*KMT2C*), which establishes histone methylation near the Wnt Family Member 5A (*WNT5A*) gene involved in dental development (odontogenesis). Syndromic forms of PFE are typically associated with complex multisystem disorders, where dental eruption failure is one of the clinical features of the spectrum. These syndromes are often linked to genetic variants that affect ectodermal development, craniofacial patterning, and skeletal growth, leading to abnormal tooth development and eruption patterns. Notable syndromes include GAPO syndrome, ectodermal dysplasia, and cleidocranial dysplasia, each contributing to PFE through distinct molecular mechanisms, such as disruptions in dental structure development, cranial abnormalities, or systemic developmental delays. The main aim of this review is to provide a comprehensive overview of the genetic basis underlying both syndromic and non-syndromic forms of PFE to facilitate precision diagnosis, foster the development of personalized therapeutic strategies, and offer new insights into managing this complex dental anomaly.

## 1. Introduction

Primary failure of tooth eruption (PFE; OMIM#125350) represents a rare yet clinically significant genetic disorder, first delineated by Proffit and Vig in 1981. Their groundbreaking work highlighted its core clinical features, such as non-responsiveness to conventional orthodontic treatment and progressive open bite, primarily affecting posterior teeth in a posterior-to-anterior pattern [1]. Despite its low prevalence of approximately 0.06%, PFE poses considerable challenges in diagnosis and management due to its complex genetic and phenotypic characteristics [2].

Tooth eruption is a multifactorial process involving intricate coordination between bone remodeling, periodontal ligament dynamics, and cellular signaling. Disorders like PFE arise from disruptions in the above-mentioned mechanisms and may have several etiologies, including local factors, such as supernumerary teeth, cysts, and tumors, as well as systemic conditions like endocrine imbalances or malnutrition [3]. However, the hallmark of PFE lies in its genetic underpinnings, specifically its autosomal dominant inheritance with variable expressivity.

The parathyroid hormone 1 receptor (*PTH1R*) gene has emerged as the primary genetic factor associated with PFE. Variants in this gene impair critical signaling pathways involved in bone and tooth development, particularly by affecting osteoclast and osteoblast functions essential for normal eruption [4]. Interestingly, the phenotypic manifestations of *PTH1R* variants vary depending on the specific genetic alteration, with some variants destabilizing the receptor’s structure, while others compromise its interactions or signaling capacity [5]. This variability underscores the importance of genetic screening, especially in familial cases, to facilitate early diagnosis and tailor therapeutic strategies.

Beyond *PTH1R*, recent studies have identified additional genetic contributors to PFE, such as Transmembrane protein 119 (*TMEM119*) and Lysine (K)-specific methyltransferase 2C (*KMT2C*). *TMEM119* variants, for instance, disrupt glycolysis-driven energy production in bone cells, leading to decreased mineralization and subsequent skeletal fragility [6]. *TMEM119*’s role in osteogenic glycolysis has been linked to specific variants that reduce the energy efficiency required for bone formation, compounding the issues of mineralization and structural integrity [7,8]. Meanwhile, genetic variants in *KMT2C*, a histone methyltransferase, highlight the role of epigenetic regulation in dental development, as they interfere with critical processes like odontogenic differentiation [9].

PFE can be clinically categorized into two distinct types: type I, characterized by the simultaneous failure of eruption across all teeth in an affected quadrant, and type II, where more distal teeth exhibit a greater eruption potential before failure [10]. Intriguingly, approximately 31% of PFE cases present with class III malocclusion, a significantly higher prevalence compared with the general population [11]. This association suggests a broader implication of skeletal and dental anomalies in the manifestation of PFE, potentially linking the disorder to disruptions in craniofacial growth and development.

The broader classification of tooth eruption disorders often includes hypodontia, which refers to the congenital absence of one to five teeth (excluding third molars) and is the most common form of tooth agenesis; oligodontia, which involves the absence of six or more teeth, representing a more severe form often linked to genetic or syndromic conditions; and anodontia, the rarest form, which is characterized by the complete absence of all primary and/or permanent teeth, typically associated with severe developmental disorders. Although these conditions may overlap with syndromic presentations, PFE’s distinct genetic etiology sets it apart from other forms of dental agenesis [12]. Notably, the differentiation of PFE from other tooth eruption failures often requires careful clinical and radiographic evaluations, particularly to exclude physical barriers or systemic syndromes contributing to the anomaly.

The study of PFE’s genetic basis has extended into syndromic contexts, exploring how overlapping genetic pathways influence both dental and systemic conditions. Genes like Periostin (*POSTN*), which encodes periostin, are being investigated for their role in non-syndromic and isolated PFE. Although causative genetic variants in *POSTN* have yet to be definitively linked, polymorphisms in its intronic regions have been observed in sporadic PFE cases, suggesting areas for further exploration [13]. Similarly, loci on chromosomes 13, 15, and 17 have been identified as regions of interest, underscoring the need for expanded genetic mapping to elucidate additional contributors to PFE’s pathogenesis [14].

**Figure 1:**
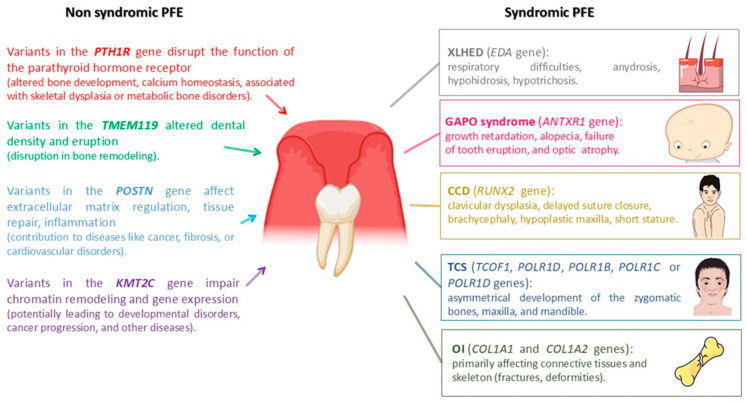
A schematic overview of non-syndromic and syndromic PFE. In non-syndromic or isolated PFE, the presence of a specific genetic variant converges toward dental agenesis or failure of tooth eruption. In syndromic forms of PFE, the genetic variant is responsible for a wide range of anomalies, including failure of tooth eruption or other dental anomalies.

This review aims at highlighting the main genes involved in non-syndromic and syndromic forms of PFE. The aim of this review is also to broaden knowledge to enhance a better precision diagnosis, foster the development of personalized therapeutic strategies, and pave the way for innovative approaches to managing PFE and related dental anomalies. Furthermore, advancing the study of PFE offers a unique opportunity to bridge molecular research and clinical practice, promoting an integrative approach to dental and craniofacial health. A schematic overview of conditions reported in this review is represented in Figure 1. PFE remains a challenging condition that requires a multidisciplinary approach, combining genetic research with clinical practice. Understanding the molecular and genetic mechanisms underlying PFE has the potential to lead to earlier diagnoses and more effective treatments.

## 2. Non-Syndromic Causes of PFE

PFE may often occur without clinical signs involving other organs or apparatuses, except for the presence of malocclusion. These forms are defined as non-syndromic or isolated PFE. In this group, genetic contributions impact crucial processes such as tooth eruption and bone mineralization. The main genes involved in isolated PFE are *PTH1R, TMEM119*, *POSTN*, and *KMT2C*, which will be discussed in detail. The findings underscore the importance of comprehensive genetic analyses in improving diagnostic accuracy and developing personalized therapeutic strategies for managing PFE. Table 1 summarizes the genes responsible for non-syndromic forms. PFE presents significant clinical challenges due to its resistance to orthodontic traction. Attempting to apply orthodontic force to the affected teeth often results in ankylosis, rendering the teeth immobile and further complicating their eruption. This can lead to functional and esthetic deficiencies, as well as long-term occlusal instability. Additionally, the inability to achieve proper eruption may necessitate complex restorative or prosthetic solutions, highlighting the importance of early diagnosis and tailored management strategies for patients with PFE [15].

### 2.1. PTH1R

The *PTH1R* gene is located in 3p21.31 and includes 16 exons. Two transcript variants encoding the same 585-amino acid protein have been found for this gene. Three promoters, P1, P2, and P3, regulate the expression of *PTH1R* [16]. The P3 promoter, proximal to the gene, seems to be turned on in many tissues and to be the most active of the three in human adult kidneys [17]. The gene encodes for the receptor for parathyroid hormone (PTH) and parathyroid hormone-like hormone (PTHLH) and has seven potential membrane-spanning domains [18]. The receptor is a member of the G-protein-coupled receptor family 2. Its activity is mediated by G-proteins, which activate adenylyl cyclase and also a phosphatidylinositol–calcium second messenger system. The expressed receptor bound PTH and PTHLH with equal affinity, and both ligands equivalently stimulated adenylate cyclase [19].

This gene is associated with several skeletal dysplasias: Murk Jansen metaphyseal chondrodysplasia (OMIM #156400), caused by activating mutations that lead to ligand-independent cAMP accumulation [20]; Blomstrand chondrodysplasia (OMIM #215045), an autosomal recessive disorder with advanced skeletal maturation and increased bone density [21]; and Eiken syndrome (OMIM #600002), characterized by delayed ossification, epiphyseal dysplasia, and bone-remodeling defects, including angel-shaped phalanges and failure of tooth eruption [22].

In 2008, Decker and colleagues [5] identified heterozygosity for two splice sites and one nonsense mutation in the *PTHR1* gene in the affected members of four German families with non-syndromic PFE that were not found in unaffected family members. The mutations were predicted to result in the premature proteolytic degradation of the precursor protein or a functionless receptor, suggesting that haploinsufficiency of *PTHR1* is likely to be the underlying principle of non-syndromic PFE [23]. PFE segregates as an autosomal dominant disorder in which non-ankylosed posterior teeth fail to move along the eruption path cleared for them, resulting in a posterior open bite [24]. The failure of affected teeth to respond to orthodontic force is a key characteristic [25]. A recent study on a cohort of 32 PFE patients demonstrated that alterations in this gene significantly impact receptor functionality, affecting protein stability, structural integrity, or gene splicing, thereby contributing to the PFE phenotype [4]. These disruptions impair the ability of the PTH1R receptor to regulate crucial processes involved in tooth eruption, highlighting its essential role in dental development [11,26]. The study emphasized that the effects of mutations in *PTH1R* vary depending on the type and position of the genetic change. For instance, certain variants may destabilize the protein structure, while others interfere with receptor interactions or compromise proper gene expression. These findings underscore the complex relationship between genetic changes and the PFE phenotype. Moreover, the research highlighted the importance of comprehensive genetic screening for patients diagnosed with PFE, particularly in familial cases. Identifying specific genetic alterations enhances diagnostic precision, allows for earlier intervention, and supports the development of personalized treatment strategies tailored to the underlying genetic causes. In conclusion, these insights provide a deeper understanding of the molecular mechanisms governing tooth eruption. By elucidating the role of PTH1R in this process, the study paves the way for potential therapies targeting these pathways to better manage PFE symptoms [20,27].

### 2.2. TMEM119

This gene, also known as *OBIF*, maps onto chromosome 12q23.3, includes two exons, and encodes for a 283-amino acid protein containing a signal sequence, a transmembrane domain, and a glutamic acid-rich region [28]. The protein has several potential O-glycosylation sites between the signal sequence and transmembrane domain, suggesting that it is a type Ia plasma membrane protein. It is involved in the positive regulation of bone mineralization; the positive regulation of osteoblast differentiation; and the positive regulation of osteoblast proliferation [29].

Xu and colleagues [2] reported two patients, a mother and her daughter, with PFE and a pathogenetic variant in *TMEM119*. Clinical evaluation revealed issues with dental eruption, accompanied by osteopenia and reduced bone density, characterized by intermittent bone pain and an increased tendency for fractures. Molecular analysis confirmed the presence of the p.(S48L) variant in *TMEM119*, which impairs the glycolytic function of bone cells and reduces their mineralization capacity [8,30]. The daughter presented with similar symptoms, including delayed dental eruption and skeletal fragility, also attributed to the p.(S48L) variant. The clinical and genetic findings of both patients indicated a condition that significantly impacts bone formation and underscores the critical role of *TMEM119* in bone mineralization processes [6,31].

The *TMEM119* gene is critical in glycolysis-mediated osteogenesis, primarily regulating energy processes in osteogenic cells. The c.143G>A point mutation results in a serine-to-leucine substitution p.(S48L) and decreases glycolytic efficiency in bone cells, thus limiting the energy required for normal bone development [8,30].

Functional studies on cultured mutant cells demonstrate that the altered TMEM119 protein exhibits reduced glycolytic activity, leading to a decreased mineralization capacity. This energy deficit impacts bone homeostasis, contributing to osteopenic conditions and increasing susceptibility to bone fragility [6,31].

### 2.3. POSTN

This gene is mapped onto 13q13.3 and includes 23 exons. It encodes for periostin, a secreted extracellular matrix protein that functions in tissue development and regeneration, including wound healing and ventricular remodeling following myocardial infarction [32,33]. The encoded protein binds to integrins to support the adhesion and migration of epithelial cells. This protein plays a role in cancer stem cell maintenance and metastasis [34,35]. Null mice exhibit cardiac valve disease and skeletal and dental defects. Alternative splicing results in multiple transcript variants encoding different isoforms. Periostin promotes cell adhesion, migration, and tumor progression via integrin pathways [36,37]. Frazier-Bowers and colleagues [25] examined families with a clinical diagnosis of PFE, focusing on specific candidate genes potentially involved in tooth eruption, such as *POSTN*, Runt-Related Transcription Factor 2 (*RUNX2*), Amelogenin-X-linked (*AMELX*), and Ameloblastin (*AMBN*). Clinical evaluations and molecular analyses were performed across multiple individuals from four families, with ages ranging from 5 to 72, highlighting patterns consistent with an autosomal dominant inheritance with variable expressivity [13]. Sequencing and mutational analysis revealed two non-functional polymorphisms in the *POSTN* gene, identified in one individual with sporadic, non-familial PFE. These polymorphisms, located in the intronic region, were noted as c.1905+27G>A and c.2100+22G>A, but did not appear to affect gene splicing or lead to functional changes. No causative variants were identified in the other candidate genes (*RUNX2*, *AMELX*, and *AMBN*) [25]. Through linkage analysis of one family a LOD score of 1.51 for marker D13S272 was obtained, suggesting that 13q21, near the *POSTN* locus, could be relevant for future investigations. Although no causative mutations were confirmed, the study highlights the potential importance of *POSTN* and nearby genomic regions in PFE with larger sample sizes and expanded family pedigrees that may help to refine the genetic basis of PFE and support the development of diagnostic and therapeutic strategies.

### 2.4. KMT2C

The *KMT2C* gene maps onto chromosome 7q36.1, a region commonly deleted in malignant myeloid disorder, and contains 60 exons spanning more than 216 kb [38]. A 1.8 kb CpG island is located in the 5′ UTR of the gene. It is a member of the myeloid/lymphoid or mixed-lineage leukemia (MLL) family and encodes a nuclear protein with an AT-hook DNA-binding domain, a DHHC-type zinc finger, six PHD-type zinc fingers, a SET domain, a post-SET domain, and a RING-type zinc finger [39] This protein is a member of the ASC-2/NCOA6 complex (ASCOM), which possesses histone methylation activity and is involved in transcriptional coactivation. KMT2C mediates the mono- and tri-methylation of histone H3 at lysine 4 (H3K4me1 and H3K4me3) [40].

In four of nine EHMT1 mutation-negative patients with core features of Kleefstra syndrome-1 (OMIM #610253) but otherwise heterogeneous phenotypes, Kleefstra et al. [41] identified variants in four functionally related genes, such as *KMT2C*, methyl-CpG-binding domain protein 5 (*MBD5*), SWI/SNF Related BAF Chromatin Remodeling Complex Subunit B1 (*SMARCB1*), and nuclear receptor subfamily 1 group 1 member 3 (*NR1I3*). All these genes encode epigenetic regulators. The variant found in *KMT2C* was a de novo heterozygous truncating mutation; this patient had a phenotype consistent with Kleefstra syndrome-2 (KLEFS2 - OMIM #617768) [42]. In five additional patients with KLEFS2, four different de novo heterozygous frameshift or truncating mutations and a de novo heterozygous 203 kb intragenic deletion in the *KMT2C* gene were found. All variants were predicted to result in a loss of function [40].

Recently, variants in this gene were identified in a family affected by an atypical form of PFE, with retention of molars and anterior teeth [9]. The clinical case concerned a four-generation family with seven affected members, showing an autosomal dominant inheritance. The diagnosis was confirmed through clinical and radiographic examinations, which revealed the presence of unerupted tooth buds with no physical barriers. To understand the genetic basis of the condition, whole-exome sequencing and whole-genome SNP analysis were performed. Genetic analysis revealed a pathogenic variant in the *KMT2C* gene, the c.1013-2A>G, causing exon skipping. The variant was considered pathogenic based on segregation analysis, its absence in the variation databases, the presence in the shared identical-by-descent region, and in silico pathogenicity prediction. Furthermore, H3 histone methylation in related genes, such as Wnt Family Member 5A (*WNT5A*), is essential for odontogenic differentiation. In summary, this variant in the *KMT2C* gene could disrupt normal tooth development, contributing to the manifestation of a dental eruption disease in this family [9].

## 3. Syndromic Causes of PFE

PFE is a complex dental anomaly frequently observed in association with other clinical signs. This review delves into the clinical features and genetic underpinnings of the most common syndromic forms of PFE, a complex dental anomaly frequently observed in association with other clinical signs. Additionally, cleft lip and/or palate is highlighted as a prevalent congenital defect in the orofacial region. Hereditary dental anomalies, including hypodontia, hyperdontia, and tooth impaction, are frequently associated with patients affected by cleft lip and/or cleft palate, occurring more commonly than in the general population. These anomalies are often linked to the complex interplay of genetic and environmental factors characteristic of these conditions. Additionally, such patients may present with asymmetries in the dental arch, malocclusions, such as crossbites, and craniofacial growth alterations, necessitating multidisciplinary treatment approaches [43]. Common syndromic forms of PFE include X-linked hypohidrotic ectodermal dysplasia (XLHED), GAPO syndrome, cleidocranial dysplasia, Treacher Collins syndrome, and osteogenesis imperfecta. By examining their distinct phenotypic presentations, this review underscores the critical role of comprehensive clinical assessment and genetic evaluation in optimizing diagnosis and management. The main clinical findings reported in syndromic forms of PFE are summarized in Table 2.

### 3.1. X-Linked Hypohidrotic Ectodermal Dysplasia (XLHED)

The EDA gene encodes ectodysplasin A, a signaling protein essential for the development of ectodermal tissues, including skin, sweat glands, hair, and teeth [44]. Ectodysplasin A functions through the EDA pathway, coordinating interactions between epithelial and mesenchymal cells that are critical for odontogenesis [45]. Pathogenic variants in the EDA gene, as observed in X-linked hypohidrotic ectodermal dysplasia (OMIM #305100), lead to disruptions in these signaling processes, resulting in characteristic dental anomalies [46,47]. Affected individuals often present with hypodontia, where some teeth are missing, or possess peg-shaped incisors and conical-shaped teeth [48]. These dental abnormalities highlight the pivotal role of EDA in regulating tooth number, morphology, and developmental patterning, underscoring its importance in ectodermal organogenesis [49].

### 3.2. GAPO Syndrome

Homozygous variants in antrax toxin receptor 1 (*ANTXR1*) have been associated with GAPO syndrome (OMIM#230740), characterized by growth retardation, alopecia, failure of tooth eruption, and optic atrophy [50].

The *ANTXR1* gene plays a pivotal role in maintaining extracellular matrix (ECM) homeostasis. Its expression has been observed in multiple tissues, including the central nervous system, heart, lungs, and lymphocytes [51]. In experimental studies on mice, targeted disruption of Antxr1 resulted in dental anomalies, such as overgrowth, incisor misalignment, and dental dysplasia [52]. These defects were attributed to ECM accumulation in the periodontal ligament and other tooth-surrounding tissues, leading to degeneration of the enamel organ and disruption of ameloblasts and odontoblasts. Interestingly, although ECM accumulation was accompanied by increased collagen type I and IV expression, no significant increase in fibroblasts was observed [51,53]. Developmental studies further demonstrated that Antxr1 is expressed at various stages of craniofacial and dental morphogenesis. Initially, the gene is localized in the epithelium and mesenchyme of developing dental structures. During later stages, its expression shifts to the polarized layers of ameloblasts and differentiating odontoblasts, highlighting its significant role in normal tooth morphogenesis. Despite these findings, the precise contribution of ANTXR1 to tooth agenesis phenotypes remains to be fully elucidated [51].

Recent analyses have challenged previous assumptions regarding complete pseudoanodontia in GAPO syndrome [54]. Instead, variants in *ANTXR1* appear to correlate with disturbed tooth eruption patterns in affected individuals. This has led to a proposed refined classification system for eruption disturbances, underscoring the importance of continuous dental monitoring for the early detection and management of potential dental developments in patients with GAPO syndrome [55]. General management strategies for PFE in GAPO syndrome may include surgical exposure of unerupted teeth, orthodontic interventions to guide eruption, and prosthetic solutions to replace non-erupted teeth, addressing the pseudoanodontia characteristic of the syndrome.

### 3.3. Cleidocranial Dysplasia (CCD)

Cleidocranial dysplasia (OMIM #119600) results from mutations in the RUNX family transcription factor 2 (*RUNX2*) gene on chromosome 6p21, a transcription factor critical for osteoblast differentiation and bone formation [56]. Variants in *RUNX2* disrupt the differentiation of mesenchymal stem cells into osteoblasts, leading to skeletal malformations [57]. Key features of CCD include clavicular dysplasia (hypoplasia or aplasia), delayed suture closure, brachycephaly, a depressed nasal bridge, hypoplastic maxilla, and short stature. Additionally, CCD frequently involves dental anomalies, such as multiple supernumerary teeth, impacted permanent teeth, and delayed eruption [58]. Supernumerary teeth, another common feature of CCD, exacerbate eruption failure by physically obstructing the eruption pathway and further complicating dental alignment. This multifactorial disruption creates a distinctive pattern of delayed eruption, impacted permanent teeth, and the retention of primary dentition often observed in CCD patients [59].

Surgical exposure of impacted teeth and orthodontic techniques may facilitate teeth eruption in CCD. PFE, frequently observed in CCD patients, poses significant challenges, requiring temporary anchorage devices and tailored strategies to enhance occlusal functionality [60].

### 3.4. Treacher Collins Syndrome (TCS)

Treacher Collins syndrome (OMIM #154500) is a craniofacial disorder marked by malar hypoplasia, downward-slanting palpebral fissures, lower-eyelid anomalies, and micrognathia or retrognathia due to symmetrical underdevelopment of the zygomatic bones, maxilla, and mandible [61,62]. Ear anomalies range from absent or malformed external ears to atresia or stenosis of the auditory canals, often resulting in conductive hearing loss due to ossicular malformations or middle-ear hypoplasia [63]. Dental and craniofacial abnormalities, such as cleft palate or choanal stenosis, play a critical role in feeding and respiratory complications during infancy. Diagnosis relies on clinical findings and molecular genetic testing for mutations in treacle ribosome biogenesis factor 1 (*TCOF1*), RNA Polymerase I And III Subunit D *(POLR1D*), and RNA Polymerase I Subunit B (*POLR1B*) or biallelic variants in RNA Polymerase I And III Subunit C (*POLR1C*) or *POLR1D* [64,65]. Typically, intellect remains unaffected, underscoring the syndrome’s isolated craniofacial impact [66]. Dental anomalies are prevalent in approximately 60% of individuals with TCS, manifesting as tooth agenesis, enamel opacities, and ectopic eruption of maxillary first molars [67]. Malocclusion, particularly angle class II with anterior open bite, is also a common feature. These dental abnormalities contribute significantly to the craniofacial and functional challenges associated with TCS, highlighting the need for interdisciplinary management involving orthodontic, prosthodontic, and surgical interventions [68].

### 3.5. Osteogenesis Imperfecta (OI)

Osteogenesis imperfecta (OMIM #166200) is a genetic disorder primarily affecting connective tissues due to mutations in the Collagen Type I Alpha 1 Chain (*COL1A1*) and Collagen Type I Alpha 2 Chain (*COL1A2*) genes, which code for the α1 and α2 chains of type 1 collagen. This protein, the most abundant in the human body, can be either structurally abnormal (qualitative defect) or produced in reduced amounts (quantitative defect) [69,70]. While skeletal manifestations, such as fractures, deformities, and chronic pain, are the most prominent features, the disease can also impact other collagen-rich organs and systems [71]. These widespread effects underscore the systemic nature of OI [72,73]. Patients with OI frequently exhibit significant dental anomalies, most notably dentinogenesis imperfecta (DI), diagnosed in approximately 25% of the studied cohort [74]. DI primarily affects individuals with moderate to severe OI and is strongly associated with qualitative collagen type 1 defects. The key features of DI include obliterated pulp chambers, shortened and thin roots, cervical constriction, and discolored crowns. Teeth affected by DI, while structurally normal in enamel, are prone to fracture, underscoring the critical role of collagen type 1 in dentine development. These findings emphasize the need for specialized dental care tailored to the severity of OI [72].

The study conducted by Futagawa explored the effect of bisphosphonates (BISs) on bone quality in children with osteogenesis imperfecta (OI), highlighting that the trabecular bone score (TBS) and bone mineral density (BMD) responded differently depending on the severity of the disease. In moderate to severe cases, TBS significantly increases with treatment duration, suggesting an improvement in bone quality. This result can be correlated with the failure of dental eruption (PFE), as bone fragility and abnormalities in mineral metabolism can also affect the development and eruption of teeth in OI patients [75].

### 3.6. Down Syndrome (DS)

Tooth agenesis is linked to a wide range of syndromes, including DS (OMIM #190685) [76,77]. Among the six studies analyzed, only one was population-based, while the others focused on specific patient groups, such as those in specialized dental clinics, potentially limiting the dataset’s representativeness. Familial hypodontia may also influence TA status in individuals with DS. A comparison with the systematic review by Palaska and Antonarakis [78], which included 1080 individuals, revealed that the TA prevalence in the present study (64%) aligns with the review’s confidence interval, with similar patterns regarding commonly missing tooth types [79].

In a study including 104 adults with DS (55 males and 49 females, aged 18–78, with a mean age of 33.8 ± 15 years), 87.5% had fewer than 25.6 permanent teeth, reflecting the mean for the general population [80]. Remarkably, 30 participants retained at least one deciduous tooth, most commonly second molars and lateral incisors [81]. Taurodontism affected 42 individuals (238 teeth), anodontia was described in 17 (44 teeth), and suspected anodontia was noted in 9 (19 teeth). Other anomalies included conic teeth (7 individuals; 11 teeth), retained teeth (5 individuals; 5 teeth), and, in single cases, root dilaceration, fusion, microdontia, and delayed eruption, totaling 84 anomalies across 329 teeth [82].

Additionally, in a separate study on German children undergoing orthodontic treatment, the prevalence of retained primary teeth (RPT) and delayed permanent tooth eruption was 59.8%. This rate may be higher in orthodontic populations than in the general population. Notably, previous dental trauma, a potential factor in RPT, was not investigated. However, a systematic review reported that ankylosis following dental trauma is rare, affecting only 1.8% of primary teeth cases [82].

## 4. Conclusions

With advancements in genetic analysis technologies and personalized therapies, significant improvements in managing this condition are achievable. A more accurate diagnosis of both syndromic and non-syndromic PFE conditions will benefit from the introduction of next-generation sequencing technologies. Multigenic panels may be more suitable for the diagnostic approach of non-syndromic PFE, while whole-exome sequencing will contribute to identifying new genes responsible for both forms of PFE. Teeth organogenesis develops through a well-ordered series of events involving genes and well-known signaling pathways, such as those of BMP, FGF, SHH, and WNT, which are able to control epithelial–mesenchymal interactions. Recent evidence indicates that more than 300 genes are involved in different phases of teeth development. Variants in genes involved in odontogenesis are responsible for many dental anomalies, including forms that can be associated with other systemic skeletal or organic manifestations (syndromic conditions) or not (non-syndromic conditions) [83]. Despite the promising advances in genetic analysis and personalized therapies, the limitations of this review include the variability in the clinical presentation of PFE and the challenge of standardizing diagnostic criteria across different populations. While the importance of early diagnosis is clear, the direct clinical implications of diagnosing PFE in its various forms remain a subject of ongoing research. However, this paper emphasizes the significance of early recognition, as it could lead to more effective, tailored treatment strategies and improved long-term outcomes for patients. Future studies should aim to better define the clinical relevance of these genetic insights, helping to refine diagnostic approaches and ultimately enhancing patient care.

In conclusion, future research in this area may lead to the development of tests for doctors to formulate an early diagnosis of these anomalies. Close collaboration among geneticists, orthodontists, and researchers would be crucial to translating these findings into practical and innovative solutions for affected patients.

## Figures and Tables

**Table 1:** List of genes responsible for non-syndromic PFE.

Gene (OMIM)	Name	Position	Function	Associated Syndromes
*PTH1R* (#168468)	Parathyroid hormone receptor 1	3p21.31	Transmembrane receptor for both parathyroid hormone and parathyroid hormone-related protein	Eiken syndrome (AR); chondrodysplasia, Blomstrand type (AR); metaphyseal chondrodysplasia, Murk Jansen type (AD)
*TMEM119* (#618989)	Transmembrane protein 119	12q23.3	Osteoblast differentiation and specific marker for microglia in the central nervous system	Not reported
*POSTN* (#608777)	Periostin	13q13.3	Cell adhesion, tissue remodeling, and angiogenesis, with roles in bone, heart development, and cancer (metastasis)	Not reported
*KMT2C* (#606833)	Lysine (K)-specificmethyltransferase 2C	7q36.1	Histone methyltransferase, which regulates gene transcription by modifying chromatin structure	Kleefstra syndrome type 2 (AD)

**Table 2:** Main clinical findings reported in syndromic forms of PFE.

Syndrome	Gene	Transmission	Tooth Anomalies	Other Anomalies
X-linked hypohidrotic ectodermal dysplasia	*EDA*	XLR	Hypodontia, with some teeth missing, peg-shaped incisors, and conical-shaped teeth	Sparse hair, inability to sweat (anhidrosis or hypohidrosis), and dryness of the skin, eyes, airways, and mucous membranes; hypohidrotic ectodermal dysplasia; and dysmorphic features
GAPO syndrome	*ANTXR1*	AR	Pseudoanodontia (failure of tooth eruption)	Ectodermal dysplasia, sparse hair (hypotrichosis), and dryness of the skin, eyes, airways, and mucous membranes
Cleidocranial dysplasia	*RUNX2*	AD	Delayed teeth eruption; supernumerary teeth	Clavicular dysplasia (hypoplasia or aplasia), delayed suture closure, brachycephaly, depressed nasal bridge, hypoplastic maxilla, and short stature
Treacher Collins syndrome	*TCOF1*	AD	Tooth agenesis, enamel opacities, and ectopic eruption of maxillary first molars	Malar hypoplasia, downward-slanting palpebral fissures, lower-eyelid anomalies, and micrognathia or retrognathia
Osteogenesis imperfecta	*COL1A1*; *COL1A2*	AD	Delayed tooth eruption, tooth discoloration (gray, brown, or bluish: dentinogenesis imperfecta), enamel fractures, and malocclusion	Hearing loss, blue sclerae, mild osteopenia, and varying degrees of multiple fractures

## Data Availability

No new data were created or analyzed in this study. Data sharing is not applicable to this article.

## Abbreviation

PFE: primary failure of eruption.
